# Drivers' performance assessment approaching pedestrian crossings through the analysis of the speed and perceptive data recorded during on-field tests

**DOI:** 10.1016/j.heliyon.2024.e24249

**Published:** 2024-01-06

**Authors:** Monica Meocci, Alessandro Terrosi, Andrea Paliotto, Roberto Arrighi, Irene Petrizzo

**Affiliations:** a Civil and Environmental Engineering Department, University of Florence, Italy; b Department of Neuroscience, Psychology, Pharmacology and Child Health, University of Florence, Italy

**Keywords:** Pedestrian crossing, Road safety, Speeding, Naturalistic driving experiment, Drivers' behavior, eye tracker

## Abstract

Pedestrian fatalities in road accidents represent one of the biggest causes of death in the world despite the great efforts that have been made to decrease the involvement of vulnerable road users in road accidents. Literature analysis revealed the presence of several studies aimed at investigating the phenomenon and proposing strategies to improve pedestrian safety, but this is still not enough to considerably reduce the number of pedestrians killed on the road.

In this context, with the aim to take a step forward in the topic, this paper describes a naturalistic driving assessment carried out in Firenze aimed at evaluating the effect of different pedestrian crossing configurations on the drivers' behavior, especially concerning the reduction of the speeding phenomenon approaching a pedestrian crossing. The experiment was conducted on a section of an urban collector road within the Firenze suburban area. Crucially, over the past few years, different traffic calming interventions have been implemented along this street. Among the different traffic calming countermeasures, both the presence of a traffic light and trapezoidal deflection have been considered to assess their effect on drivers' behavior, also with reference to specific aspects related to the drivers’ perception. During the experiment, thirty-six users drove their own vehicles along the street, encountering different pedestrian crossing configurations. During the driving speed, deceleration and ocular fixation were recorded.

This study shows the difference in drivers' behavior in response to different traffic calming countermeasures. It demonstrates also that the raised pedestrian crossing caused a significant effect on reducing the speed approaching a pedestrian crossing. Moreover, it is observed that, when perceptive countermeasures are present, the drivers’ behavior changes only if the pedestrian crossing configuration is perceived in foveal vision; suggesting that the correct identification of the configuration is crucial to implement a congruent and safe driving behavior.

## Highlights

• Improvement in pedestrian safety represents one of the most important challenges across the world.
• Speed control in urban areas represents one of the main safety problems to be solved. The on-field experiments conducted give suggestions to mitigate speeding phenomena in relation to drivers' behavior approaching pedestrian crossings.
• A statistically significant decrease in the speed approaching pedestrian crossing were obtained when the trapezoidal deflection is present.
• A statistically significant increase in the driver's attention was observed when the trapezoidal deflection is present.

## 1 Introduction

In recent years, crash data has been strongly affected by mobility restrictions due to the COVID pandemic. However, both worldwide and national reports concerning road accidents confirm the trends of previous years, with the highest number of accidents being recorded in urban areas [1].

In particular, in Italy, the last ISTAT report regarding traffic collisions occurred in 2021 and states that 73.1 % of total crashes occur in urban areas. These crashes involve 43.9 % of total fatalities and 69.7 % of injured people [2]. The percentages are no different from those recorded in the last year before the pandemic, where accidents, deaths and injuries in urban areas were respectively 73.7 %, 41.9 % and 69.9 % [3]. The percentage of pedestrian deaths remained equal in Italy, and it is approximately 16 % of the total road deaths, these values are lower than the European average equal to 21 % [4]. Data concerning the pedestrian mortality is very different across Europe, and it takes on minimum values in Northern Europe, where dead pedestrians account for approximately 1/4 of the deaths recorded in Italy (e.g., Netherland, Swedish, etc.). The maximum values instead are reached in the Eastern Europe (e.g., Romania), where deaths exceed more than 3 times the EU average [5].

The preliminary report on US roads stated that 2022 represents the worst year for pedestrian deaths since 1981 and that deaths of pedestrians surged 19 % in the last three years [6]. Statistics show a serious issue all over the world.

An analysis of the Italian historical accident data (2014–2018) conducted by Meocci et al., in 2021 [7] revealed that 30 % of traffic collisions involving pedestrians occur at pedestrian crossings, which should be the safest place for pedestrians that are crossing the street. Therefore, it is necessary to seriously understand the factors affecting the phenomenon and provide effective solutions that reverse the trend and mitigate this issue worldwide.

Up to this moment the topic has been widely evaluated through different methodologies and different perspectives, both in terms of the pedestrians' behavior [8,9] and the drivers’ behavior. In the latter case, the speed at which drivers approach areas where vehicle-pedestrian interaction is frequent, represents the main contributory risk factor for crash probability and severity, both due to the increase of collision force and reduction in users' reaction time [[10], [11], [12], [13], [14], [15], [16], [17], [18]]. Therefore, to reduce the risk of pedestrian collisions, interventions capable of mitigating excess speed were frequently highlighted as a starting point [19,20].

A possible approach to research this topic is the passive approach, which is based on the analysis of crash database in order to identify of both crash-prone locations and factors affecting the phenomenon [7,[21], [22], [23], [24]]. However, this approach fails to go into detail and does not investigate the crash environment or the road characteristics approaching the site of the crash [25] and often does not consider driving speed as a variable. Moreover, the crash databases are often inaccurate or contain data characterized by imprecise location description (e.g., “*near to the church*”, “*closer to the intersection*”, etc.) or only referred to a specific type of accident (e.g., completely missing in property damage only crashes), thus losing a very high number of occurrences.

Other studies instead investigate the collision in a proactive manner, through direct investigation of the driver-pedestrian interaction using traffic conflict-based techniques [[26], [27], [28]] or driving simulators [16,29,30]. Driving simulator research investigated the attitudes and behaviors (especially with reference to the drivers) in pedestrian-drivers’ interaction (e.g., approaching pedestrian crossing). These types of studies allow to evaluate different scenarios also characterized by different safety countermeasures to reduce the number of conflicts and interactions [16,30,31].

Unfortunately, the scenarios are often unable to reproduce the real world, and thus the results could be biased or not entirely representative of participants real behavior.

Naturalistic driving studies thus represent the most reliable methodology to evaluate both behavior and performance of different road users during their interaction with each other. This approach allows to investigate the road users' behavior in a natural environment; and has been defined by the European Transport Safety Council as “*research studies to find out more about driver behavior on daily trips through recording details of the drivers, vehicle, and environmental performance by using types of equipment and without control devices*” [25,32]. Naturalistic driving allows therefore to evaluate road users' behavior in different real road sections “as a test section” without any traffic restriction (if possible). Here both the effect of different road configurations and safety countermeasures can be evaluated through a direct analysis of the users' behavior. In recent years numerous studies have tried to evaluate drivers’ behavior in response to different pedestrian crossing or road configurations [[33], [34], [35]].

In 2013 Habibovic et al. stated that to propose effective road safety countermeasures, it is necessary to understand how and why safety-critical situations and/or crashes occur. Therefore, they carried out research where 90 video recordings of car-to-pedestrian conflict were assessed by onboard cameras in a naturalistic driving study in Japan, demonstrating that crashes with similar car trajectories can be grouped and the causation were similar and too often identified as obstruction on the road [36].

Jurecki and Stanczyk in 2018 evaluated driver response to a pedestrian that crosses the street (from the left or right) by a naturalistic driving study conducted with a sample of 30 drivers. The finding highlighted that the driver's reaction time changes in face of different critical situations [37].

In 2018 Lin et al. [37] conducted a naturalistic driving study to investigate drivers’ compliance with selected pedestrian features at signalized intersections. The research highlighted that driver compliance could be improved through the countermeasures analysed in the presence of pedestrians and that young drivers, older drivers, female drivers, and risky drivers tend to have lower probabilities of compliant behavior.

Sheykhfard et al. [38] assessed vehicle-pedestrian interaction through naturalistic driving studies using fixed and in-motion cameras. In 2021 the same author used a similar approach to analyze crosswalks safety and surrogate safety indicators [25]. Research made it possible to highlight that the drivers’ behavior is essential to avoid a collision, especially in high-severity collisions.

However, while the aforementioned studies only recorded the driving behavior in terms of vehicle telemetries, a deeper insight into the driver's behavior and perception would provide a deeper understanding of the phenomenon.

In the last years, naturalistic driving research has been improved thanks to the use of eye tracker devices coupled with black boxes or other devices able to record video and speed data of the car during the driving session. This approach solves the inability of the on-board camera to record the gaze and the factors affecting the visual perception of the drivers. The eye-tracker records the movement of drivers' pupils to understand if the environment (e.g., road, road features, light condition, people near the road, etc.) can affect the drivers’ reaction and behavior [39]. Naturalistic driving studies with eye-tracker devices were conducted to understand the crash-causing factors [40], the effect of road signs and advertisements on driver attention [41] and acceleration/deceleration behavior [[42], [43], [44]] or the road safety performance [45].

In terms of pedestrian crossing safety, the first research investigating the drivers' behavior approaching pedestrian safety by eye-tracker devices was conducted in virtual reality. Ciceri et al., in 2013 [46] evaluated the effect of pedestrian crossing configuration and pedestrian obstruction on the driver reaction. The study found a correlation between environmental complexity and the driver's reaction time. Similar research evaluated the effect of marking and crosswalks configuration on the drivers' perception and reaction approaching pedestrian crossing [47,48].

The use of an eye-tracker on on-field tests regarding pedestrian safety and crosswalks allowed Vignali et al. [49] to conduct a before-after analysis on a new pedestrian crossing configuration (absence/presence of the intervention). The research demonstrates the effect of road marking on drivers’ perceptions. The same authors, in 2020 conducted new research regarding different treatment and traffic calming measures addressing pedestrian safety [50].

In 2021, Babic et al. [51] use the eye tracking to analyze the pedestrian conspicuity at night, investigating how the drivers perceive pedestrians wearing different clothes (black or light-colored clothes, or an orange or yellow retroreflective vest) at different distances. This study analyses a large sample of driving and also defines a consistent analysis methodology. Another recent study concerning road safety use the eye-tracking devices in order to evaluate the effect on driver distraction due to the advertisement [52]. Driver distraction was also investigated trough the eye tracking devices by many other authors (e.g., Refs. [53,54]).

In this context, the need to improve pedestrian safety, especially at pedestrian crossings and the possibility of conducting on-field tests that specifically analyze how traffic calming countermeasures based on drivers' perceptions affect the drivers' reaction and consequently change the drivers’ behavior approaching pedestrian crossing emerged.

## 2 Research objectives

As already highlighted in the introduction, the pedestrian safety assessment, with reference to different configurations of pedestrian crossings, has been extensively analysed in virtual reality but remains only poorly investigated through on-field tests.

Literature shows different pros and cons both for virtual reality and naturalistic driving studies [32,55]. While virtual reality evaluations are characterized by safe and cheaper environments, naturalistic driving experiments are characterized by a high-quality data able to describe the real-world events in a reliable manner [32]. In behavioral analysis this represents a good starting point to improve the description of drivers' behavior while they are driving within an uncontrolled environment. (e.g., real traffic conditions, real road users’ interactions, weather effect on driving behavior, etc.).

In these terms, to evaluate the drivers’ response to different engineering treatments it was decided to conduct an experimentation within a suburban area of the city of Firenze. Via Pistoiese was selected as the experimental site firstly because of its high rate of pedestrian accidents and secondly because of the presence of both different pedestrian crossing and traffic calming measures recently realized.

The street was classified as an urban collector road, and it occupied the second place for accidents involving pedestrians in Firenze. Pedestrian safety in via Pistoiese has been recently improved through a reconfiguration project of many pedestrian crossings and intersections designed on the basis of perceptual principles. Currently, different pedestrian crossing configurations are present, both traditional and experimental (i.e., zebra crossing, humps, signalized pedestrian crossing, etc.).

The main objective of this paper is therefore to compare the basic configuration of crosswalks (i.e., zebra crossing) with alternative configurations characterized by the presence of traffic lights and trapezoidal deflection (raised crosswalks) to define how the pedestrian crossing configuration affects the drivers' behavior, and if they improve pedestrian safety by decreasing speeds [34,[56], [57], [58]], according to the Human Factor principles. The use of specific equipment recently acquired allows to introduce some innovative opportunities to conduct safety assessment aimed at carried out a more in-depth investigation of the effect of the drivers' perception in their behavior and aggressiveness. This approach defines the basis for the introduction in the research of a preliminary assessment aimed at improving Vulnerable Road Users safety by using and evaluating countermeasures that directly change the drivers’ behavior by means of cognitive and perceptive process in order to mitigate the speeding phenomena in urban area.

In this context, this paper describes an advanced procedure to assess how perceptual countermeasures allow to mitigate the speeding phenomena. The analysis considers driving indicators (e.g., speed, acceleration, etc.) as the main factors, but also includes a preliminary evaluation of drivers’ perception by the eye-tracking introduction. This last point constitutes the starting point for future research, given in this paper only qualitative but innovative information.

## 3 Experimentation

### 3.1 Case study in Firenze

#### 3.1.1 Case study selection

In the 2014–2018 period 2059 fatal or injuries accidents involving pedestrians occurred in the urban area of the municipality of Firenze.

**Fig. 1:**
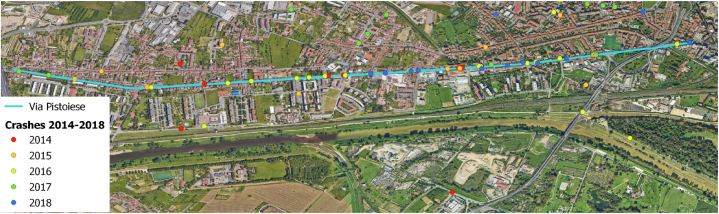
Location of accidents in Via Pistoiese differentiating by colour per year. (For interpretation of the references to color in this figure legend, the reader is referred to the Web version of this article.)

In the five-year period, 45 accidents occurred in Via Pistoiese, making it one of the most dangerous roads of the road network of Firenze. Fig. 1 shows the location of the accidents that occurred in the period 2014–2018, with different colors representing different years of occurrence.

Due to the high number of crashes here occurred, past research, according to the Road Strategic Plan of the Firenze Municipality, identified the need to reconfigure the whole area to improve the vulnerable road user's safety [29,59]. The project introduces along the road a set of traffic calming measures aimed mainly at limiting driver's speed [60] and also to improve the spaces where the presence of pedestrians is high, with specific reference to areas of engaging between pedestrians and motor vehicles, such as pedestrian crossings. These safety interventions, extensively described in Ref. [29] and completely realized between 2017 and 2018, configured the street as a very particular section within the city of Firenze, where traditionally configured areas alternate with experimental solutions. Therefore, because of the presence of different safety solutions implemented along the street, it was decided to consider via Pistoiese as a test section and consequently carry out an on-field experimentation in the overall area.

#### 3.1.2 Case study description

Via Pistoiese is an urban road in the municipality of Florence whose main function is to connect the western suburban areas with the city center. According to art. 2 of the Highway Code [61] it can be classified as a type E road, i.e., “urban collector road”.

**Fig. 2:**
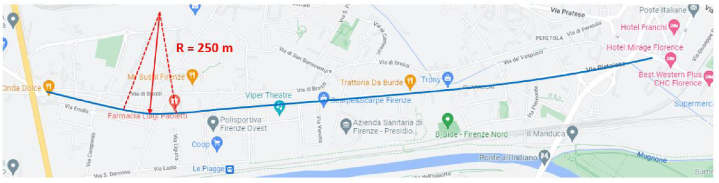
Overview of the road axis of Via Pistoiese.

The street starts at the roundabout near the A1 motorway overpass and ends at the intersection with Via Baracca. The road axis consists of two long straight connected by a circular curve (R = 250 m) and has a total length of about 3.8 km (Fig. 2). From the altimetric point of view, the road is almost flat.

**Fig. 3:**
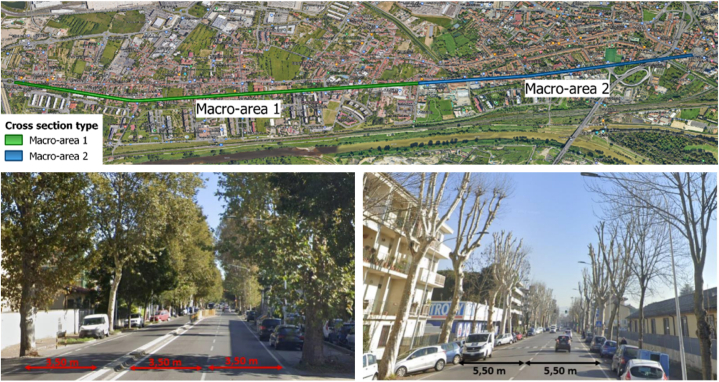
a: view of the two macro-areas; b: organization of the road section of Via Pistoiese in macro-area 1; c: organization of the road section of Via Pistoiese in macro-area 2.

Looking at the cross-section, Via Pistoiese can be divided into two different macro-areas (Fig. 3a).

● the macro-area 1 (the most suburban) has a section with a total width of 18.5 m and includes three lanes, alternating 2 + 1 lanes in both directions to allow the correct circulation of traffic during peak hours. Each lane is 3.5 m wide. The two directions are divided by a curb that can be drove-over by emergency vehicles. There are also two lateral areas 2 m wide generally used for both authorized and sometimes illegal parking, and two sidewalks 1.5 m wide (Fig. 3, b);
● the macro-area 2 (the one closest to the city center), has two traffic lanes 5.50 m wide not physically divided from each other (Fig. 3, c); the 2 m space for parking and the sidewalks of 1.5 m are also present.

The macro-area 2 is 1.5 km long, while the macro-area 1 is 2.3 km. The speed limit is 50 km/h for both areas described, according to the provisions of the Highway Code [61] for “urban collector road” (type E).

On both sides of Via Pistoiese there are densely populated residential areas and numerous commercial activities. In addition, numerous intersections, pedestrian crossings, and driveways (e.g., private access road) are present along the road to allow access to the commercial and residential areas.

**Fig. 4:**
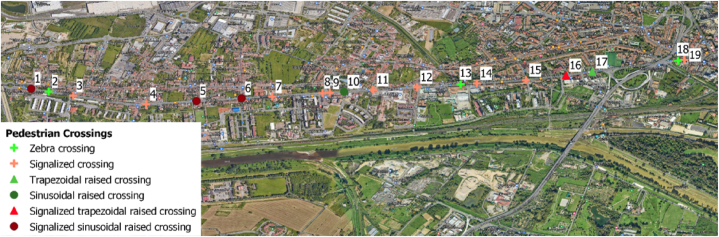
Location of pedestrian crossings.

Analysing the pedestrian crossings along the road, a remarkable heterogeneity in the types can be observed. Table 1 summarizes all the pedestrian crossings of Via Pistoiese indicating their type, while Fig. 4 shows their locations.

**Table 1:** Configuration of pedestrian crossing in Via Pistoiese.

ID	Configuration of pedestrian crossing
2, 13, 18	Zebra crossing
3, 4, 7, 8, 9, 11, 12, 14, 15, 19	Signalized crossing
17	Trapezoidal raised crossing
10	Sinusoidal raised crossing
16	Signalized trapezoidal raised crossing
1, 5, 6	Signalized sinusoidal raised crossing

#### 3.1.3 Pedestrian crossings analyses

The crosswalks analysed were chosen to investigate how different pedestrian crossing configurations can affect human perceptions and thus human behavior, both in the perceptual domain (what is fixed or not) and in the driving domain (speed profile).

It should be noted that depending by changing the direction of traffic the geometric configuration, for instance, the number of lanes (i.e., 2 lanes in one direction and 1 in the other), and the road configuration before the crossing, may be significantly different (i.e., distance to other crossings and intersections). Therefore, the pedestrian crossings were categorized and analysed twice, once for each direction of traffic. In addition, pedestrian crossings within macro-area 1 were characterized by narrower lanes if compared with those in macro-area 2 (3.5 m vs 5.5 m wide) which is separated by a surmountable curb.

The types of pedestrian crossing were then categorized as follows, for each of which the identification codes of the crossings belonging to the type under consideration are given, accompanied by a letter indicating the direction of travel considered (i.e., I=Inbound, O=Outbound, with reference to the center of Firenze).

**Fig. 5:**
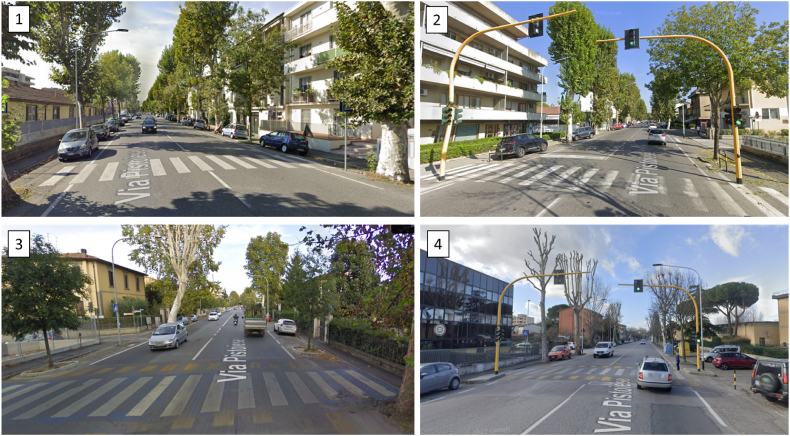
Pedestrian crossing configuration analysed (1: zebra crossing, 2: signalized zebra crossing, 3: trapezoidal raised crossing, 4: signalized trapezoidal raised crossing).

1. zebra crossings with one wide lane in the direction of travel: crossings 13-I and 13-O fall into this category (Figs. 5–1);
2. signalized zebra crossings with one wide lane in the direction of travel: crossings 14-I, 14-O and 15-I fall into this category (Figs. 5–2);
3. trapezoidal raised crossings with one wide lane in the direction of travel: crossings 17-I and 17-O fall into this category (Figs. 5–3);
4. signalized trapezoidal raised crossings with one wide lane in the direction of travel: crossings 16-I and 16-O fall into this category (Figs. 5–4).

Fig. 5 represents the configuration of those analysed, which are the pedestrian crossing listed in the previous bullet point. It has been chosen to consider only those crossings where the driver's behavior is not influenced by other elements close to the crossing (e.g., other pedestrian crossings and intersections). Therefore, only crossings with more then 150 from the previous and next element have been considered.

## 4 Methodology

### 4.1 Ethical and participant information

Before describing the participants and detailed the experimental procedure, authors would like to highlight that for experimentations like the one presented in this paper, the University of Florence suggests but does not require the mandatory approval of the ethics committee. Therefore, this test did not collect the opinion of the ethical committee, which is now mandatory required by the University only for tests involving humans but concerning medical experiments.

However, when the recruitment of participants was carried out, EXCLUSIVELY on a voluntary basis, each participant was advised about the testing methods and their opportunity concerning the experimentation, by signing an informative consent.

The informative consent form was divided into two parts.

1. the first in which personal data was collected, and where it was explained that the only use of the personal data was the scheduling of the tests. This data (name and surname of each participant) would only be stored until the end of the experimentation. Then, all participant personal information were anonymized by means of an alphanumeric code for the analysis and post-processing of data collected and results elaboration and exploitation. Participants were also informed that no pictures or video showing their person were recorded during the test.
2. the second part was instead related to the test methodology. In this section authors clearly stated the procedure, and that each participant could renounce or interrupt his test at any time, without giving justification.

To give the maximum protection of the participants during the test, additional insurance was taken out on the number plates of all cars used by each participant during the test, additionally to the mandatory one required for their own cars. This was to cover any kind of inconvenience, which fortunately did not occur.

### 4.2 Apparatus

The on-field experimentation was conducted to evaluate the drivers’ behavior approaching pedestrian crossings by combining data coming both from eye-tracking measurements and driving behavior measurements.

**Fig. 6:**
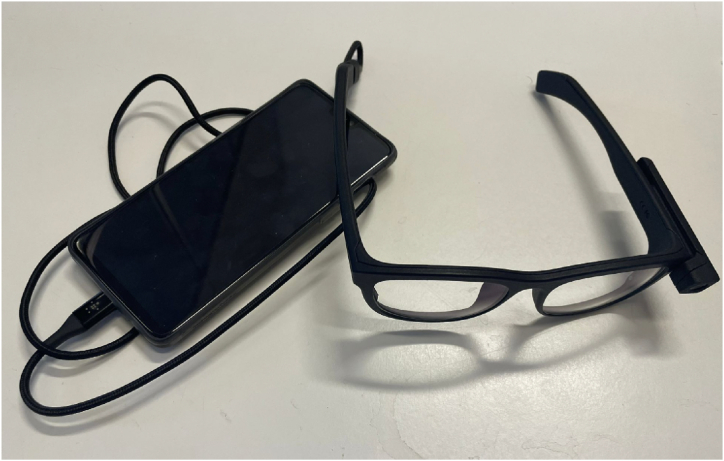
Pupil Invisible eye-tracker.

The Pupils Invisible, a portable eye-tracker mounted in the innerframe of transparent non-corrected glasses, was used to record eye-tracking data (Fig. 6).

**Fig. 7:**
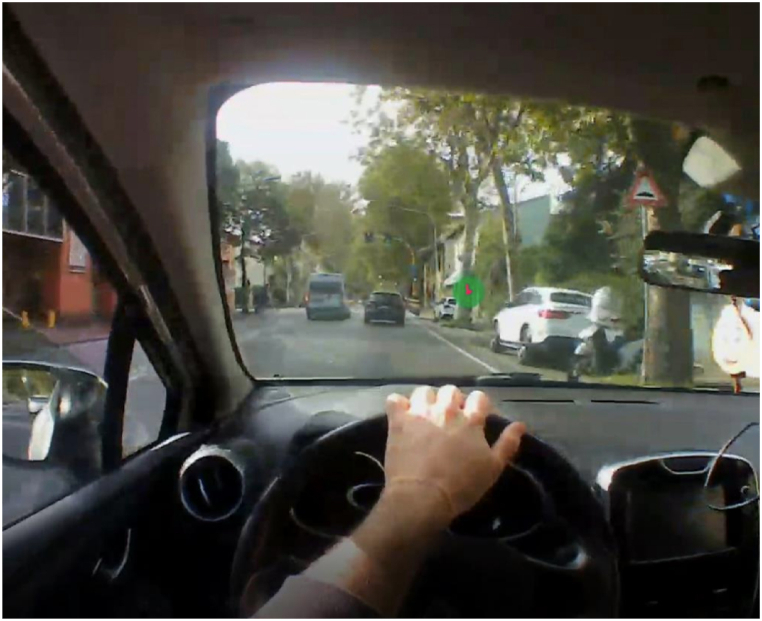
Example of a frame with the driver's gaze.

Three video cameras are fixed to the glasses. Two of the three cameras allow the detection of the eye pupils and their movement. These are plotted on the “world” video recorded by the third one (Fig. 7) with a frame rate of 30 Hz. The device is connected to a smartphone that allows the experimenter to both observe the experimentation in real-time and to manage the recorded video. By being extremely portable and non-invasive, this device allows capturing the eye movements of the drivers with high ecological value which can then be used to analyze driver's saccades and fixations.

**Fig. 8:**
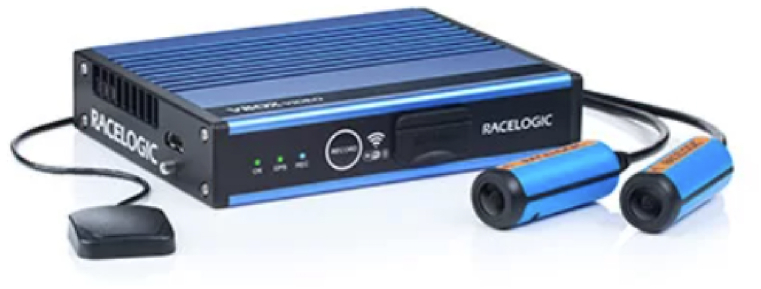
Vbox HD2 (https://www.vboxmotorsport.co.uk/index.php/en/products).

To acquire kinematics data about the drivers’ performance (i.e., speed, acceleration, position, etc.) A VBOX HD2 system (Fig. 8) was used to acquire kinematics data about the drivers' performance (i.e., speed, acceleration, position, etc.). The VBOX continuously records the GPS position of the vehicle with a frame rate of 10 Hz. It is also able to record two videos with two HD cameras (1080-pixel resolution) whose view and orientation were defined by the user/operator. All the data recorded can be exported by.csv file and remotely analysed.

### 4.3 Participants

Thirty-six subjects (28 men and 8 women, mean value: 40.6 years; standard deviation: 17.2 years) were recruited on a voluntary basis among students at the University of Firenze (Italy), experienced drivers, and external people. All participants had normal or corrected-to-normal vision with the use of contact lenses.

The statistical sample was constructed in such a way that there was a good percentage of expert drivers: 12 participants (33 %) have more than 10 years of driving license and travel an average of more than 20,000 km per year. Furthermore, young users (15–34 years), characterized also by a number of driving license years less than 10 (17 participants, 47 % of the sample), or elderly users (over-55 years), characterized also by a number of driving license years more than 25 (9 participants, 25 % of the sample), were preferred. These criteria in the sample selection aim to analyze the driving behavior of the classes of users considered most at risk of accidents, i.e., young and elderly people. The former as they have less driving experience and tend to drive more aggressively and the latter as they have less reactivity and less capacity in terms of perception.

Almost all the sample (92 %) declared that they had travelled on all types of existing roads (urban, extra-urban and motorway routes) in the last 12 months. Furthermore, this percentage rises to 97 % of the total sample considering those who travel urban-type routes daily. Finally, except for 5 users, all of them declared that they travelled at least 5000 km in a year.

### 4.4 Procedure

Each participant was tested individually according to the following procedure.

**Fig. 9:**
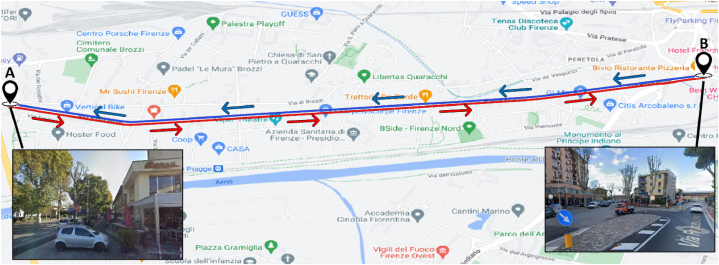
Test path (in red inbound, in blue outbound). (For interpretation of the references to color in this figure legend, the reader is referred to the Web version of this article.)

3. *Participant instructions*: each participant was instructed on the test procedure but not on the research objectives then they were instructed on the test route to follow (point 4) during which they must never switch off the engine until the operator had given their consent, to be able to correctly save the recording. So, they were invited to have driving behavior as faithful as possible to what they ordinarily practice. And again, to ensure natural behavior, each participant was asked to drive their own vehicle to eliminate as much as possible the conditioning due to driving a different/unusual vehicle.
4. *Mounting the VBOX HD 2 device in the vehicle*: the device was positioned near the gearbox to reduce the eccentricity between the device and the vehicle's center of gravity, whereas the GPS was fixed externally to the vehicle in the upper part of the front right-side door.
5. *Wearing and calibrating eye tracker*: each volunteer wore eye-tracker glasses; the device was connected to the mobile phone via cable, and it was checked that the system was working properly. Therefore, the calibration phase was conducted on the specific user.
6. *Test route*: once the above operations were completed, the participant starts driving along the chosen route, which is shown in Fig. 9. Starting from the meeting point (A), fixed in the car park located near the roundabout where Via Pistoiese begins, participants drove on Via Pistoiese up to the intersection with Via Baracca, following the red route. From here, after a short break, the participants were asked to carry out the reverse path (blue route in Fig. 9), reaching the point from which the experiment started.

The whole procedure lasted around 30 min, of which 10 min for the preliminary operations (1, 2 and 3) and 20 min for the driving phase (4). All tests were carried out outside of peak traffic hours, to avoid as much as possible intense traffic conditions which can affect the drivers’ performances (i.e., speed) and behavior.

### 4.5 Data collection and analysis

According to the research objective, the analysis of the data resulting from the naturalistic driving experimentation focused on two main categories of parameters.

1. **Driving indicator**: data related to the drivers' speed profile approaching the different configurations of the pedestrian crossings analysed; and
2. **Perceptual indicator**: data related to the effect of the environment and road configuration in driver perception and their influence on driver behavior.

The driving indicators included.

● the mean speed profile approaching in the 150 m before the pedestrian crossing
● the drivers' speed at the pedestrian crossing axis (V_z_);
● the drivers' speed at the stopping sight distance (V_stop_) to define the ability of the driver to stop the vehicle in case to unexpected events. Here a speed limit of 50 km/h was considered to define about 54 m of stopping sight distance;
● difference in speed approaching pedestrian crossing (DV) if a decelerating behavior was observed;
● distance from the crosswalks' axis and the beginning of the deceleration phase (D).

The perceptual indicators included.

● drivers' gaze approaching pedestrian crossing. The pupils' movements were evaluated in reference to the drivers' behavior previously analysed (e.g., difference in speed). Here, the fixation was analysed as a parameter describing the drivers' ability to recognize a specific configuration affecting their behavior.

The presence of signalized crosswalks strongly affected the speed profile approaching each pedestrian crossing, especially when the traffic light was red. Therefore, all data concerning red traffic lights were eliminated. Unfortunately, the eliminated data also contain events where pedestrians crossed the street, but in controlled environment (red traffic light for vehicle). Only a few times during the experimentation (4–5 times) pedestrians were crossing the street with green light or on unsignalized crosswalks arising a vehicle-pedestrian interaction. In terms of analysis, the number of interactions was therefore very low to give consistent results and then evaluate the occurrence in terms of “typical driver behavior”. Consequently, all the data concerning pedestrians crossing were eliminated from the analysis.

A preliminary outliers’ detection procedure was conducted to understand whether there were outliers on the data set with reference to the first (Q1) and third (Q3) inner quartiles. Only the data falling below the Q1 value (i.e., describing traffic congestion or specific traffic condition that can affect the drive of the volunteer) were considered outliers. Then, the remaining data were statistically analysed in terms of mean value and standard deviation. The sample size was 253.

The drivers' behaviors were analysed considering the four (4) dependent variables: Vz, Vstop, DV and D. To analyze the relationships among dependent variables the MANOVA test was suggested to provide insights into the patterns of association and interactions among multiple outcomes measures. However, because of the small sample size obtained and the violation of different hypotheses required for the MANOVA test, in this research, a two-way ANOVA test was performed to define whether there were statistically significant effects on the dependent variable describing drivers' performances as a function of the pedestrian crossing configurations, providing only insights into specific effects on individual outcomes but lacking the broader perspective. Two independent variables describing crosswalk configuration were selected: traffic light and trapezoidal deflection. Each variable was characterized by two levels: presence and absence of the safety countermeasures (i.e., traffic light: presence/absence and trapezoidal deflection: presence/absence). Further analysis with Bonferroni adjustment was carried out to evaluate the pairwise combinations of the within-subject factors. A preliminary Levene's test was performed to assess the equality of variances (homoscedasticity).

The ANOVA test on the four dependent variables indicated above (Vz, Vstop, DV and D) was then repeated to evaluate the effect (if present) of both the age and driver experience.

In the end, the two-way ANOVA was repeated to test if the drivers’ gaze significantly affects the driving indicator approaching a pedestrian crossing. This analysis was conducted only with reference to the drivers who had a fixation on the pedestrian crossing markings. The sample size was reduced to 133.

General and final consideration and analysis were finally conducted to combine the overall results obtained, also in a qualitative way.

## 5 RESULTS and DISCUSSION

### 5.1 Mean speed profile

**Fig. 10:**
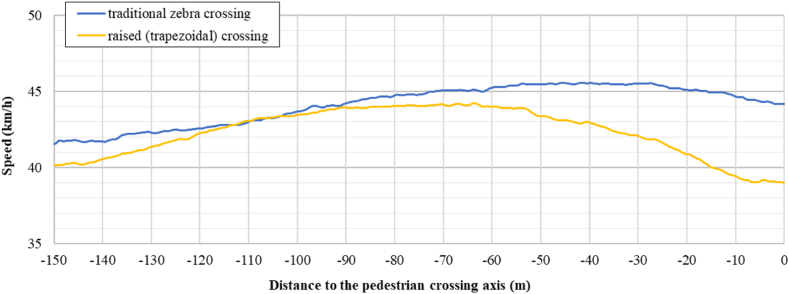
Speed profile approaching zebra/raised crossing.

**Fig. 11:**
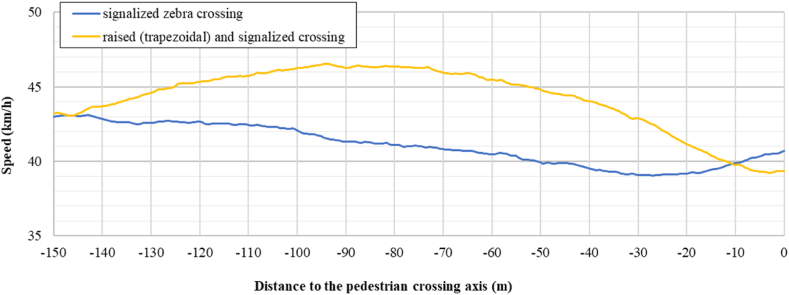
Speed profile approaching signalized zebra/raised crossing.

Fig. 10, Fig. 11 show the mean speed profiles obtained for the four crosswalks configurations analysed. Fig. 10 describes the mean speed profile of the drivers while approaching a pedestrian crossing without a traffic light, while Fig. 11, describes the speed profile approaching a pedestrian crossing characterized by the presence of a traffic light. It is evident from the two plots that the speeds on the axis of the crosswalk were less than 40 km/h. However, when the vertical deflection was absent the speeds are higher and seems to be not affected by the presence of the pedestrian crossing.

An immediate observation can be made in terms of generic behavior.

● no decelerations (or very low) were recorded for traditional zebra crossing (traffic lights or not);
● acceleration behaviour was recorded in reference to the signalized zebra crossing; and
● high decelerations were recorded for trapezoidal raised pedestrian crossings (traffic lights or not). In this last case, deceleration is observed starting from about 70 to 80 m before the axis of the pedestrian crossing.

The results obtained are aligned with the main literature findings [17,29,30,35]. Due to the prevailing presence of the male gender in the participant sample (78 %) the results mainly reflect the male driving behavior.

### 5.2 Speed describing drivers’ behavior approaching pedestrian crossings

The analysis conducted on the drivers' reaction approaching pedestrian crossings (evaluated with reference to the axis position) showed that the crosswalk configuration had a small effect on the drivers’ speed approaching crossings.

Results obtained from the preliminary driving indicators, concerning both the V_z_ and V_stop_ were summarized in Table 2.

**Table 2:** Preliminary results obtained in the experimentation.

Configuration^a^	“1”	“2”	“3”	“4”
V_z,mean_ (km/h)	44	41	39	39
V_z,SD_ (km/h)	7.62	7.26	6.54	5.74
V_z,max_ (km/h)	61	57	58	60
V_z,min_ (km/h)	31	26	27	31
N (V_z_ > 50 km/h)	12	9	3	2
V_stop, mean_ (km/h)	46	40	44	45
V_stop,SD_ (km/h)	6.96	7.29	7.41	6.32
V_stop,max_ (km/h)	68	57	59	67
V_stop,min_ (km/h)	34	26	31	36
N (V_stop_>50 km/h)	21	12	7	5

a “1”: zebra crossing; “2”: signalized zebra crossing; “3”: trapezoidal raised crossing; “4”: signalized trapezoidal raised crossing.

The raised pedestrian crossing (trapezoidal with/without traffic light) caused a lower speed on the pedestrian crossing axis. The comparison of the V_z_ values referred to the presence/absence of traffic lights seems to be negligible, especially when it was considered coupled to the trapezoidal deflection. Furthermore, lower standard deviations were recorded; then, the drivers’ behavior can be described as “more homogeneous” in the presence of traffic calming countermeasures than in the traditional zebra crossing configuration.

The number of users who exceed the speed limit in correspondence with the axis of the pedestrian crossing decreases from 12 to 2. Consequently, the data shows that the presence of the raised pedestrian crossing influences (at least qualitatively) the drivers' behavior. The V_stop_ instead, does not follow the same principles and there aren't specific speed trends, however, also in this case, the number of users exceeding speeds decreases considerably for raised crossings, if compared to traditional zebra crossings. But in this case, the differences in V_stop_ were considerable if referred to the configuration “1” and “2” (zebra crossing without/with traffic light).

In Table 3 the drivers’ behavior was described both in terms of deceleration measured (as the difference in V (if) occurred approaching pedestrian crossing) and distance (D) where a change in the speed profile is evident.

**Table 3:** Drivers’ behavior described by deceleration.

Configuration^a^	“1”	“2”	“3”	“4”
DV_mean_ (km/h)	7.41	11.17	9.02	10.04
DV_SD_ (km/h)	4.38	6.00	3.69	4.77
%	32	20	51	56
D_mean_ (m)	52.79	76.52	65.87	72.63
D_SD_ (m)	24.91	31.20	22.03	28.28

a “1”: zebra crossing; “2”: signalized zebra crossing; “3”: trapezoidal raised crossing; “4”: signalized trapezoidal raised crossing.

According with the technical/scientific literature, results obtained showed that the DV considerably increases if the crossing is raised [35,[62], [63], [64]]. The percentage of drivers that showed a deceleration behavior doubles if the pedestrian crossing is raised. Instead, the traffic light allows anticipating the start of braking, despite the green color.

The described results don't include pedestrians crossing the street when the vehicle is passing. This is a consequence of the small number of “crossing events” in the recorded data. Therefore, the results obtained are representative of everyday driving conditions and not of the interaction between pedestrians and vehicles.

To quantify the phenomenon described, a two-way ANOVA test was conducted to determine if there are statistically significant interaction effects on the selected dependent variables due to the pedestrian crossing configurations described by two independent variables (traffic light and deflection), each one characterized by two levels: presence or absence of countermeasures. In Table 4 the results were summarized.

**Table 4:** ANOVA test results: pedestrian crossing configuration effect on dependent variable.

**Independent variable**	Dependent variable							
	Vz (km/h)		Vstop (km/h)		DV (km/h)		D (m)	
	*F*	*p-value*	*F*	*p-value*	*F*	*p-value*	*F*	*p-value*
*Traffic light*	3.510	0.062	5.968	**0.015**	0.766	0.382	7.937	**0.006**
*Trapezoidal deflection*	8.060	**0.005**	3.397	0.067	30.201	**<0.001**	0.413	0.522
Traffic light*trapezoidal deflection	6.428	**0.012**	11.047	**0.001**	1.156	0.283	2.069	0.153

The preliminary Levene's test for homogeneity of variance interpretation was violated only in the first analysis (V_z_); however, the ANOVA test is robust and even if this hypothesis was violated the result is supposed to be sufficiently reliable. In the other analysis conducted (V_stop_, DV and D), Levene's test confirmed the homogeneity assumption of the variance.

The results of the two-way ANOVA test revealed a significant main effect (level of significance equal to 5 %) of the trapezoidal deflection with reference to the V_z_ and DV. Therefore, a significant decrease in speed depends on the pedestrian crossing configuration, especially when a trapezoidal deflection is present. The independent variable traffic light instead has a significant main effect both in V_stop_ and D. The interaction effect was found only for V_z_ and V_stop_.

The pairwise comparison of interaction effects with Bonferroni adjustment revealed that V_z_ was significantly lower (equal to 38.6 km/h) when the trapezoidal deflection was present (V_z_ mean difference = 2.716 km/h and p = 0.005). Instead, the dependent variable V_stop_ was significantly lower (39.6 km/h) when the traffic light was present (V_stop_ mean difference = 3.264 km/h and p = 0.015).Furthermore, the same analysis revealed that DV was significantly higher (6.8 km/h) when the trapezoidal deflection was present (mean difference 3.860 km/h and p < 0.001), while D was significantly higher when the traffic light was present (mean difference 13.796 and p = 0.006).

The results obtained confirmed the importance of the countermeasure selected in decreasing speed approaching the pedestrian crossing and revealed that the high visibility of the traffic light allows drivers to pay attention to their speed (results were also confirmed by the pairwise comparison referred to V_stop_ and D). In terms of V_z_ and DV results revealed that the deflection affects the drivers’ behavior on the pedestrian crossing probably because of the effect of “speed bump” on their vehicle if correctly perceived and recognized.

**Fig. 12:**
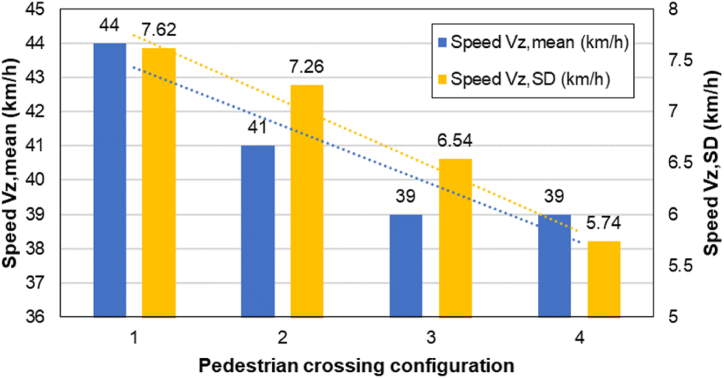
Speed and speed variance at different configuration of pedestrian crossing (“1”: traditional zebra crossing; “2”: signalized zebra crossing; “3”: raised (trapezoidal) crossing; “4”: signalized and raised (trapezoidal) zebra crossing).

In Fig. 12 the significative speed decrease in correspondence of the crossing axis (V_z_) was shown (in light blue) as a function of the crosswalk's configuration. Furthermore, in grey is indicated also the evident decrease of the speed variance as a function of the crosswalk's configuration. Already in 1998, Migletz et al. [65] stated that the safest traffic flow occurs when all drivers travel at the same speed (approximately); thus, the higher the speed variance the higher the risk of a crash. The graph demonstrates the effect on the safety of the countermeasures evaluated in these terms (speed control and reduction).

In conclusion, the result obtained in the on-field test demonstrates the validity of the countermeasure to uniformly decrease the speed and increase road safety. The most effective countermeasure was represented by the trapezoidal deflection, but also the traffic light allows to improve road safety. When the trapezoidal deflection was present drivers decreased their speed by about 5 km/h, equal to 13.9 % of the speed before the deceleration. Considering instead only the drivers who had a deceleration behavior, the mean DV was about 8 km/h, i.e., 19.6 % of the initial speed. Similar results are obtained by Yeo et al. [35]. Conversely, the results obtained by Mohammadipour et al. [62] and Gitelmant et al. [63] indicate more effectiveness of the countermeasures. The speed reduction sometimes reached 50 %. The two countermeasures coupled instead do not justify, in terms of safety improvement, a higher intervention cost but they affect in different ways parameters related to a safety drivers’ behavior.

The two ANOVA tests conducted to evaluate the significance of both the age and driver experience didn't highlight significant effects on the dependent variables considered. Therefore, contrary to the statistics which identify more aggressive driving behavior in young people, data collected in the on-field experimentation did not highlight significant trend in young people behavior.

### 5.3 Drivers’ gaze approaching pedestrian crossings

The previous analysis shows that the number of drivers who showed a decelerating behavior double if the trapezoidal deflection is present. With reference to these results, a more in-depth analysis was conducted to determine the main factor affecting the driver's deceleration according to the driver's path of eye fixation while approaching the pedestrian crossing. This approach represents a new and preliminary activity to introduce in our research at least one parameter allowing the description of how drivers act subsequently the observation of the road section and their elements.

Contrarily to the expectations, the first results obtained showed that only a small number of users fixed their gaze on the vertical signs; therefore, no specific analysis was conducted for this item. Instead, but also obviously, the main target of drivers’ fixations were traffic lights at intersections. Moreover, and contrarily to the vertical signs, markings were frequently observed especially approaching pedestrian crossings.

Consequently, two categories of factors were directly investigated: traffic lights and pedestrian crossing markings. In Table 5 the gaze analysis was summarized.

**Table 5:** Drivers’ gaze analysis.

Crossing Types	Variable observed	
	Traffic light	Pedestrian crossing marking
*Zebra crossing*	–	58 %
*Signalized zebra crossing*	72 %	51 %
*Trapezoidal raised crossing*	–	85 %
*Signalized trapezoidal raised crossing*	69 %	83 %

The results allow us to observe that the pedestrian crossing located on a trapezoidal hump is the one that most attracted the attention of the drivers' gaze. Specifically, the drivers’ fixations were directed at the yellow and black signs indicating the presence of the hump ramp. Qualitatively, it can be observed also that the percentage of markings observations on the pedestrian crossing decreased if there is also a traffic light on the crosswalk. No significative difference in traffic light observations were recorded in the two different pedestrian crossings analysed.

To quantify the significance (or not) of the drivers' gaze and perception in reducing the speeding phenomena, a two-way ANOVA test was repeated only considering the drivers who had a fixation on the pedestrian crossing recognizing the crosswalks’ type (zebra or raised). This choice was made with reference to the ability of the eye-tracker device to record the fixation, i.e., where the driver is locating his foveal vision for visual coding and information processing [66]. In these terms, since the process of recognizing a specific road configuration (e.g., the presence of humps) is a complex task, it can only be performed if the driver analysed the context in foveal vision and not in peripheral vision. Consequently, all the drivers who have not had fixations at the pedestrian crossing have either not found it interesting or have not seen it at all.

The analysis was then repeated with a reduced sample (133 vs 253 drivers) considering all the four dependent variables, V_z_, DV, V_stop_ and D. Table 6 summarized the result obtained.

**Table 6:** ANOVA test results: driver gaze effect on dependent variable.

**Independent variable**	Dependent variable							
	Vz (km/h)		Vstop (km/h)		DV (km/h)		D (m)	
	*F*	*p-value*	*F*	*p-value*	*F*	*p-value*	*F*	*p-value*
*Traffic light*	1.402	0.239	1.652	0.201	0.832	0.363	2.015	0.160
*Trapezoidal deflection*	4.627	**0.033**	2.218	0.139	15.167	**<0.001**	2.853	0.096
Traffic light*trapezoidal deflection	2.778	0.098	3.752	0.055	0.365	0.547	0.205	0.652

The analysis confirmed that only the trapezoidal deflection (if observed in foveal vision) caused a significant effect both in the decrease of the speed on the pedestrian crossing axis (V_z_) and in the increase of the deceleration (DV) with respect to the initial drivers' speed. Conversely, there are no other elements which, if perceived by the driver, cause a significant change in drivers’ behavior. According to the different percentage of drivers who fixed the different pedestrian crossing it can be stated that the drivers did not consider of interest to elaborate the information connected to the zebra crossing, which therefore does not have salient elements or elements which indicate the driver to have specific driving behaviors (or decelerations), which instead are caused when the deflections are present.

**Fig. 13:**
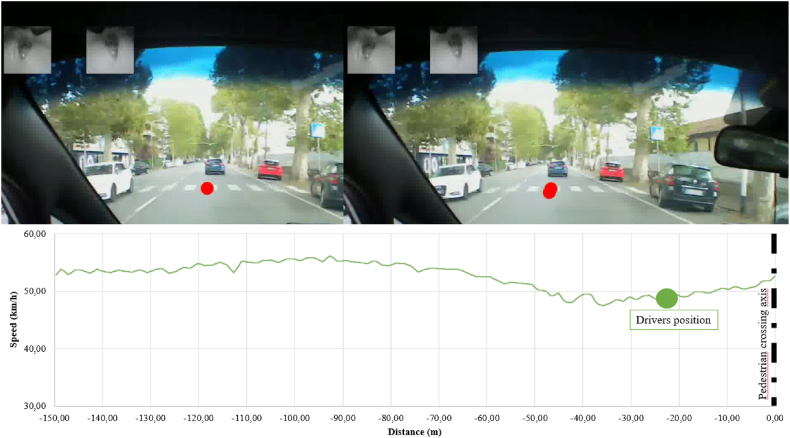
Driver's behavior approaching zebra crossing.

**Fig. 14:**
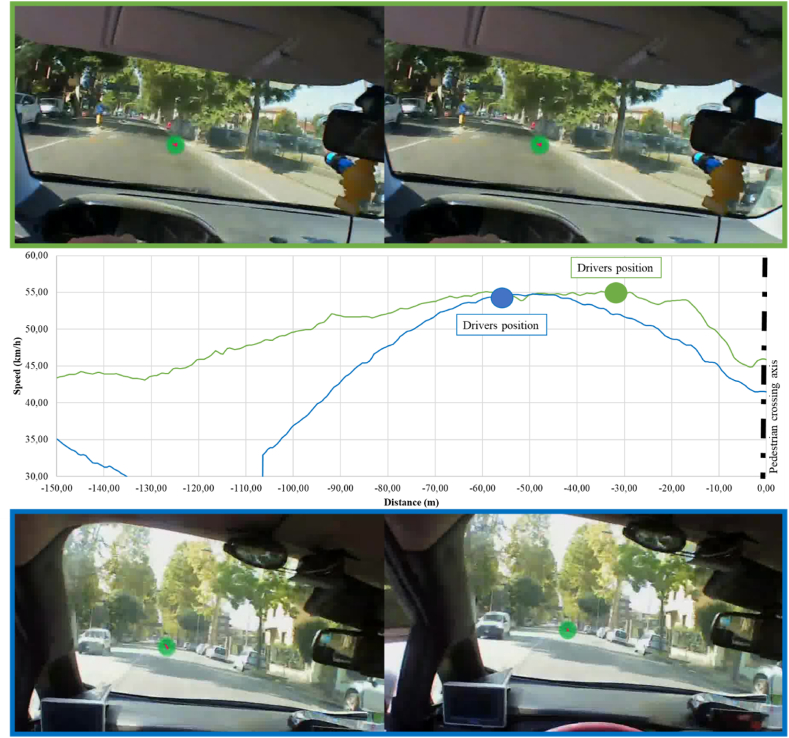
Driver's behavior approaching trapezoidal deflection (two different drivers approaching the same trapezoidal raised crossing).

Fig. 13, Fig. 14 allow observing the difference in the drivers’ behaviors with reference to the approaching traditional zebra crossing (absence of safety countermeasures) and trapezoidal deflection. In the first case (Fig. 13) the driver had a fixation on the zebra crossing; however, no elements that affected the need to decelerate are present. Therefore, the driver accelerates independently of the crossing presence, contrary to the behavioral rules defined by the Italian Highway Code [61] in these road configurations.

When trapezoidal deflections are present (Fig. 13), instead the driver who fixed the pedestrian crossing is forced to decelerate in order not to have “dynamic effects on his own vehicle” even in the absence of pedestrians crossing, demonstrating an increase in safety thank to the reduction of speeding phenomena.

In conclusion, the on-field analysis carried out highlights the effectiveness of perceptual measures, such as raised pedestrian crossings, in reducing the speed when approaching the pedestrian crossing. The study of perceptive factors using eye-tracker devices allows confirm the results obtained by emphasizing that the safety measure for having “gaze fixations” have to be salient in the environment, and, only in this occurrence, the driver will adjust its behavior to the recognized situation.

The analysis conducted on the driving experience did not provide statistically significant results but made it possible to qualitatively observe differences in some trends i.e., higher speed in certain pedestrian crossings for young drivers or delayed perception in the face of the same configuration for those over-55 (e.g., green VS blue speed profile in Fig. 14).

This last part of the paper represents a step forward to improve the research methodology with new technologies but gives only preliminary results and information about the influence of the perception on the phenomenon and driving tasks. Moreover, the analysis constitutes the starting point for future analysis focused on more precise evaluation of the effect of perception on road safety.

## 6 Conclusion

An extensive on-field experimentation was conducted along via Pistoiese, one of the most accident-prone streets in Firenze partly due to the speeding phenomena. This particular road was chosen as, in recent years the street was interested in a reconfiguration project aimed at reducing driver speed, and then improving overall road safety, especially for vulnerable road users, in order to reduce the pedestrian-accident issues.

To investigate how the different pedestrian crossing configurations affect the drivers' responses and speed, driving parameters and driver's eye movements were recorded.

The comparative analysis of the pedestrian crossings selected allowed to demonstrate the following findings.

● A significant decrease in the speed approaching pedestrian crossing when the trapezoidal deflection is present. The two-way ANOVA test demonstrated the effect of the trapezoidal deflection both in decreasing the speed on the axis of the pedestrian crossing and increasing the value of deceleration. In fact, in the latter case, the deceleration is double the average one recorded in the zebra crossings.
● The traffic light does not improve the drivers' performance in response to a pedestrian crossing but improves the visibility of the area, therefore, when a green light is present the speed at a stopping sight distance is significantly lower than in the other conditions.
● A significant increase in the drivers' attention on the pedestrian crossing markings when the trapezoidal deflection is present. Drivers who had a fixation on the pedestrian crossing increased from 58 % to 85 % if the pedestrian crossing is trapezoidal.
● The fixation on the crossing is significant in recognizing the traffic calming measure, therefore in having the right behavior (deceleration) in the face to the safety countermeasures. It has been shown that only drivers who look at the trapezoidal deflection have a high deceleration. Conversely, the users who do not show fixations on the traffic calming measure do not show deceleration behavior because they cannot recognize the effective road configuration (i.e., estimate the deflection high and possibly armful to the vehicle).

The results obtained are in line with the existing literature and defined the trapezoidal deflection as an effective countermeasure to improving pedestrian safety by a significant decrease in speeding phenomena.

The use of eye-tracking in field experiments represents an innovative procedure for improving road safety research methodology, especially for understanding the effect of perceptual and cognitive countermeasures (e.g., traffic calming, colored areas, chicanes, etc.). In this work, the results obtained are preliminary and form the starting point for future analyses focusing on a more precise assessment of the effect of perception on driver behavior.

Though the very simple analysis conducted on the gaze evaluation, it allows to have an important result from the experimentation: none of the participants ever fixated on the vertical signs. Therefore, to increase the drivers’ attention, vertical signs should either be moved to a more central field of vision (for example, up through portals) or become brighter (i.e., such as traffic lights).

In terms of perceptive measures, as the behavior of the driver depends strongly on the countermeasure visibility, it is necessary to carry out frequent maintenance interventions in order not to compromise the cognitive-perceptive phenomenon which determines the recognition of the road section configuration and therefore the proper reaction in terms of driving behavior (i.e., deceleration).

In conclusion the study suggests introducing trapezoidal deflection in the pedestrian crossing configuration to mitigate the speeding phenomena and to pay attention on signs and marking position and maintenance. The methodology for driver gaze analysis needs to be improved and deepened in future research with the aim of obtaining more in-depth information on how perception affects driving tasks and drivers’ behavior.

## Funding

The SWALK research was funded by the Università degli Studi di Firenze, call “Finanziamento di progetti competitivi per Ricercatori a Tempo Determinato (RTD) dell'Università di Firenze”, 2020–2021.

## Data availability

Data associated with the research has not been deposited into a publicly available repository but can be obtained upon request.

## Additional information

No additional information is available for this paper.

## CRediT authorship contribution statement

**Monica Meocci:** Writing - review & editing, Supervision, Project administration, Methodology, Funding acquisition, Data curation, Conceptualization. **Alessandro Terrosi:** Writing - original draft, Software, Formal analysis, Data curation. **Andrea Paliotto:** Writing - review & editing, Writing - original draft, Investigation, Formal analysis. **Roberto Arrighi:** Writing - review & editing, Project administration, Funding acquisition. **Irene Petrizzo:** Writing - review & editing, Methodology, Investigation.

## Declaration of competing interest

The authors declare that they have no known competing financial interests or personal relationships that could have appeared to influence the work reported in this paper.
